# Formulation and Characterization of Patient-Friendly Dosage Form of Ondansetron Hydrochloride

**DOI:** 10.4103/0975-1483.66796

**Published:** 2010

**Authors:** PK Bhoyar, DM Biyani, MJ Umekar

**Affiliations:** *S.K.B. College of Pharmacy, New Kamptee, Dist. Nagpur, Maharashtra, India*

**Keywords:** Indion 234, taste masking, sodium starch glycolate, ondansetron hydrochloride, orodispersible tablet

## Abstract

Ondansetron hydrochloride is an intensely bitter antiemetic drug used to treat nausea and vomiting following chemotherapy. The purpose of the present work was to mask the taste of ondansetron hydrochloride and to formulate its patient-friendly dosage form. Complexation technique using indion 234 (polycyclic potassium with carboxylic functionality) and an ion-exchange resin was used to mask the bitter taste and then the taste-masked drug was formulated into an orodispersible tablet (ODT). The drug loading onto the ion-exchange resin was optimized for mixing time, activation, effect of pH, mode of mixing, ratio of drug to resin and temperature. The resinate was evaluated for taste masking and characterized by X-ray diffraction study and infrared spectroscopy. ODTs were formulated using the drug–resin complex. The developed tablets were evaluated for hardness, friability, drug content, weight variation, content uniformity, friability, water absorption ratio, *in vitro* and *in vivo* disintegration time and in vitro drug release. The tablets disintegrated in vitro and in vivo within 24 and 27 s, respectively. Drug release from the tablet was completed within 2 min. The obtained results revealed that ondansetron HCl has been successfully taste masked and formulated into an ODT as a suitable alternative to the conventional tablets.

## INTRODUCTION

Convenience of administration and patient compliance are gaining significant importance in the design of dosage forms. Recently, more stress is laid down on the development of an organoleptically elegant and patient-friendly drug delivery system for pediatric and geriatric patients.[1,2] More than 50% of the pharmaceutical products are orally administered for several reasons, and undesirable taste is one of the important formulation problems encountered with such oral products.Taste of a pharmaceutical product is an important parameter for governing compliance. Thus, taste masking of oral pharmaceuticals has become an important tool to improve patient compliance and the quality of treatment, especially in pediatrics. Therefore, formulation of taste-masked products is a challenge to the pharmacists.[3,4]

Ondansetron HCl is a potent antiemetic drug indicated for the treatment and/or prophylaxis of postoperative or chemotherapy- or radiotherapy-induced emesis, and is also used in the early onset of alcoholism.[5] In general, emesis is preceded with nausea and, in such a condition, it is difficult to the administer drug with a glass of water. Hence, it is beneficial to administer such drugs as orodispersible tablets (ODTs). Ondansetron HCl is an intensely bitter drug; hence, if it is incorporated directly into an ODT, the main objective behind formulation of such a dosage form will definitely be futile.[6] Thus, in the present study, an attempt has been made to mask the taste of ondansetron HCl and to formulate ODTs with a good mouth feel so as to prepare a “patient-friendly dosage form.”

Mouth-dissolving tablets are dosage forms that disintegrate in patient’s mouth within a few seconds without the need of water, or chewing, providing best remedy for patients suffering from dysphagia. Their growing importance was underlined recently when the European pharmacopoeia adopted the term “orodispersible tablet” as a tablet that is to be placed in the mouth where it disperses rapidly before swallowing.[7]

Ion-exchange resins have been increasingly used as taste-masking agents. They are also known to be useful as disintegrating agents.[8,9] Thus, the study undertaken was aimed at using ion-exchange resins for both the purposes, formulating taste-masked ODTs of ondansetron HCl.

The complex of cationic drug and weak ion-exchange resin does not break at the pH of saliva, i.e. 6–7, with a cation concentration of 40 meq/l. But, at the high cationic concentration in the stomach and pH 1.2, free drug is immediately released. This implies that while passing through the mouth, the drug remains in the complex form, thereby imparting no bitter taste in the mouth. This property was exploited to formulate the “consumer-friendly dosage form,” i.e. mouth-dissolving tablets.[10]

## MATERIALS AND METHODS

### Materials

Ondansetron HCl was a gift sample from Alkem Laboratories (Mumbai, India). Indion 234 was obtained from Ion Exchange India Ltd., Mumbai, India. Starlac and MCC (PH 101) were provided by SignetChemicals Ltd., Mumbai, India. All other chemicals and reagents used were of high analytical grade.

### Methods

#### Preparation of drug–resin complex (resinate)

Resinate were prepared using the batch method.[11] Water is used as a medium for complexation because the drug is soluble in water.[12] An accurately weighed amount of resin (100 mg) was placed in a beaker containing 50 ml of deionised water. Accurately weighed 100 mg of ondansetron hydrochloride was added to the resin solution and stirred for 180 min. The mixture was filtered through a Whatman filter paper and the residue was washed with 50 ml of deionised water to remove any uncomplexed drug. Unbound drug in the filtrate was estimated at 310 nm and the drug-loading efficiency was calculated.

#### Optimization of Ondansetron HCl–indion resin complexation

The drug loading on to the resin was optimized for various parameters such as mixing time, activation, effect of pH, mode of mixing, ratio of drug:resin and effect of temperature.

#### Optimization for mixing time on drug loading

Separate batches of indion 234 (100 mg) were soaked in 50 ml of distilled water in a beaker and about 100 mg of drug was added and stirred for 3 h. The mixture was filtered through a Whatman filter paper and the residue was washed with 50 ml of deionized water to remove any uncomplexed drug. Unbound drug in the filtrate was estimated at 310 nm and the time required for maximum adsorption of the drug was optimized.

#### Effect of activation of resin on drug loading

Resins were washed with distilled water and subsequently with 1 N HCl. The resins were rewashed with water until neutral pH was reached. Drug:resin complexes were prepared by placing 100 mg of acid-activated resins in a beaker containing 50 ml distilled water and about 100 mg of drug and stirred for 3 h, and the drug content was determined as mentioned previously. Similarly, alkali activation of indion 234 was performed, replacing 1 N HCl with 1 N NaOH.

#### Effect of pH, mode of mixing, ratio of drug:resin and temperature on drug loading

For optimization of pH, weighed, 100 mg of drug was added to 100 mg of activated resins in 50 ml of distilled water. The pH of the solutions was adjusted at 3.0, 3.5, 4.0, 4.5, 5.0, 5.5, 6.0 and 6.5 and stirred for 3 h and the drug content was determined as mentioned previously.[11] For optimization of mode of mixing, rotary shaker and magnetic stirrer were used. For all activated resins (100 mg) in 50 ml of distilled water and about 100 mg of drug, the pH was adjusted at 3.5 and the drug content was determined as mentioned previously. For optimization of ratio of drug:resin, three batches were prepared containing drug–resin in the ratio of 1:1, 1:2 and 1:3. The pH was maintained at 3.5. The solution was stirred using a magnetic stirrer for 3 h. To study the effect of temperature, separate batches were prepared containing drug–resin in the ratio of 1:3. The pH was maintained at 3.5 and was stirred using a magnetic stirrer at 30°, 35°, 40°, 50° and 60°C and the drug content was determined as mentioned previously.

#### Characterization of resinate

FT-IR spectrum of the drug, resin and resinate were recorded over the wave no 4000 to 400 cm^-1^ on a Jasco Dispersive type FT-IR spectrophotometer using the KBr disc technique. Then, the spectra were analyzed to determine the formation of complex of the drug and resin.

The drug, resins and resinate was subjected to X-ray diffraction study for the confirmation of complex formation. X-ray diffraction studies were carried out on a Phillips analytical X-ray BV (pw1710) using Cu anode 40 kv voltage and 30 mA current.

#### Determination of drug content

Resinate equivalent to 8 mg of drug was stirred with 100 ml of 0.1 N HCL for 3 h, till the entire drug leached out, and then the solution was filtered through a Whatman filter paper. Further dilutions were made with 0.1 N HCl and the drug content was determined spectrophotometrically at 310 nm using 0.1 N HCl as blank.

#### Taste evaluation of solid drug:resin complex

The drug–resin complex (1:3) was subjected to sensory evaluation by a panel of nine members using the time intensity method. The pure drug without complexation with the ion-exchange resin was used as control in this study. Sample equivalent to 8 mg (dose of drug) was held in the mouth for 10 s. Bitterness was recorded instantly and then after 20, 30, 40, 50 and 60 s. The evaluation was performed by classifying bitter taste into five levels: level 0: no bitter taste is sensed; 1: acceptable bitterness; 2: slightly bitter; 3: moderately bitter; 4: strongly bitter. Descriptive statistics, mean and standard deviation were calculated for all variables. Paired *t*-test was applied using INSTAT software. Value *P*<0.05 has been considered as the statistical significance level.

#### In vitro dissolution

Complexes of Ondansetron hydrochloride with indion 234 were subjected to *in vitro* dissolution studies using the USP 24 method. Weigh quantity of complexes equivalent to the normal dose and suspended in 0.1 N HCl using the USP II dissolution apparatus. The quantity of drug released was determined periodically.

#### Formulation development

Resinates of drug (dose of drug 8 mg) were formulated into tablet by the direct compression technique. using sodium starch glycolate, microcrystalline cellulose (PH-101) and spray-dried mixture of starch and lactose (Starlac). Each formulation was composed of drug and excipients in various proportions, as shown in Table 1. All ingredients were passed through mesh no. 60. The required quantity of each was taken for the particular formulation and the blend was mixed using a mixer. Powder blend was evaluated for micromeritic properties like shape, angle of repose, bulk density, tapped density and Housner ratio.[13,14] Mixed blend of drug and excipients was compressed on an 8-station rotary punch tablet machine (Karnavati, India). Tablets, each weighing 150 mg, were prepared.

**Table 1:** Formulation design

Time (s)	Before taste masking (mean ± SD)	After taste Masking (mean ± SD)
10	4.0 ± 0.00**	0.3 ± 0.44**
20	3.2 ± 0.50**	0.09 ± 0.33**
30	2.4 ± 0.52**	0
40	2.0 ± 0.50**	0
50	1.7 ± 0.44**	0
60	1.22 ± 0.44**	0

* Containing 8 mg of drug

#### Evaluation of tablets

The prepared tablets were evaluated for various official and nonofficial specifications.[15,16] Twenty tablets were selected at random and the average weight was calculated. Then, individual tablets were weighed and the individual weight was compared with an average weight. Tablets were evaluated for hardness and friability testing using the Monsanto hardness tester and the Roche friabilator, respectively. For drug content uniformity, 20 tablets were weighed and crushed. The resinate powder was weighed to get 8 mg drug equivalent and transferred to 100 ml of 0.1 N HCl and shaken for 15 min and centrifuged. Further dilutions were made with 0.1 N HCl and the drug content was determined spectrophotometrically at 310 nm using 0.1 N HCl as blank.

#### Water absorption ratio

A piece of tissue paper folded twice was placed in a small Petri dish containing 6 ml of water. A tablet was put on the tissue paper and allowed to wet completely.[16] The wetted tablet was then weighed. Water absorption ratio, R, was determined using the following equation:

$$R\;=\;100\;\times \;\left\{W_{a}\;-\;W_{b}\right\}/W_{b}$$

Where, W_b_ = Weight of tablet before water absorption

W_a_ = Weight of tablet after water absorption

#### In vitro dispersion time

The tablet was put into 100 ml distilled water at 37°C ± 2°C. Time required for complete dispersion of a tablet was measured with the help of a digital tablet disintegration test apparatus.

#### In vivo dispersion time

*In vivo* dispersion time of a tablet was checked in healthy human volunteers by putting a tablet on the tongue and measuring the time required for complete dispersion of a tablet.

#### Dissolution study

Tablets formulated with resinates were subjected to *in vitro* dissolution studies using the USP type II apparatus (Paddle type) at 100 rpm with a temperature of 37°C ± 0.5°C. Dissolution was carried out in 0.1 N HCl as the dissolution medium. After a specific time interval, 5 ml of the dissolution medium was withdrawn by a pipette and replaced with fresh medium for maintaining the sink condition. The sample was filtered and absorbance of the filtered solution was determined by UV spectroscopy at 310 nm. Dissolution rate was studied for all designed formulations and the conventional marketed tablet.

## RESULTS AND DISCUSSION

### Optimization of Ondansetron HCl–Indion 234 complexation

Ondansetron was loaded onto Indion 234 by the batch process. Complexation is essentially a process of diffusion of ions between the resin and the surrounding drug solution. As reaction is an equilibrium phenomenon, maximum efficacy is best achieved in batch process.

Complexation between drug and resin increases up to the optimum time and then remains almost constant. It was found to be optimum after 3 h of mixing in the resin investigated. Highest drug binding on the resin was achieved when activated with 1 N HCl.

The percentage drug loading with inactivated resin, treated with acid and alkali, was found to be 45.55 ± 1.6% w/w, 54.61 ± 0.5 and 49.65 ± 0.11% w/w, respectively. After activation with acid treatment, the exchangeable ion on the resin is H^+^. Relative selectivity of H^+^ is least than other ionic forms and therefore it increases the percent complexation. The mode of complexation between drug and resin can be affected by pH of the media. Maximum drug loading on the resin occurs at pH 3.5; a maximum of 71.79 ± 0.73% w/w for indion 234. As pH increases above 3.5, percentage of drug loading decreases. pH of the solution affects both solubility and degree of ionization of the drug and resin. Results can be attributed to the fact that a cationic drug is ionized at lower pH value and hence demonstrates high binding capacity while at higher pH, the protonated fraction of cationic drug decreases and hence interaction with resin also decreases.[11] Hence, Ondansetron HCl as a cationic drug will have maximum solubility and complete ionization in this range. Decreased complexation at lower pH, i.e. below 2, is due to excess H^+^ ions in solution, which have a higher binding affinity to the –COO group of the resin and compete with the drug for binding. Complexation was found to be optimum when using a magnetic stirrer, a maximum of 71.79 ± 0.73% w/w for indion 234 and, in case of a rotary shaker, a maximum of 65.27 ± 0.54% w/w. This finding may indicate the significant involvement of van der Vaal’s forces taking place along with drug exchange during complexation. The drug loading in various drug:resin concentrations was found to be 71.79 ± 0.73% w/w, 78.24 ± 0.86% w/w, 83.82 ±0.57% w/w for 1:1, 1:2 and 1:3 ratio, respectively. Increase in drug resin ratio above 1:3 did not further increase the percentage drug loading. The % drug loading (w/w) with temperature of 30°, 35°, 40°, 50° and 60°C was found to be 76.82 ± 0.23% w/w, 83.82 ± 0.57% w/w, 88.52 ± 0.53% w/w, 93.87 ± 0.36% w/w and 93.34 ± 0.19% w/w, respectively. These figures reveal that as temperature increases, the percentage of drug loading also increases rapidly up to 50°C. Increase in temperature above 50°C did not further increase the percentage drug loading. Increased temperature during complexation increases ionization of the drug and resin. Higher temperatures tend to increase the diffusion rate of ions by decreasing the thickness of the exhaustive exchange zone. Also, at increased temperatures, swelling of the resin takes place. Due to swelling, the ionic sites are open for exchange of counter ions.

The drug content in the resinate was found to be 98.55%. The dissolution profile of the drug showed complete drug release within 90 s. Results of evaluation of taste indicated complete masking of the bitter taste as no bitterness was felt in the drug–resin complex [Table 2].

**Table 2:** Volunteers’ opinion test for Ondansetron HCl before and after taste masking by a panel of nine members using the time intensity method (n = 9)

Ingredients	Formulations							
	B1	B2	B3	B4	B5	B6	B7	B8
Indion 234 resinate (mg)	9*	9	9	9	9	9	9	9
Sodium starch glycolate (mg)	----	----	4.5	6	7.5	4.5	6	7.5
Starlac (mg)	111	----	106.5	105	103.5	----	----	----
Avicel (PH 101)	----	111	----	----	----	106.5	105	103.5
Fructose	25	25	25	25	25	25	25	25
Aspartame	1	1	1	1	1	1	1	1
Magnesium stearate	2	2	2	2	2	2	2	2
Aerosil	2	2	2	2	2	2	2	2
Orange flavor	QS	QS	QS	QS	QS	QS	QS	QS
Total (mg)	150	150	150	150	150	150	150	150

*P*<0.001**

#### Characterization of resinate

**Figure 1:**
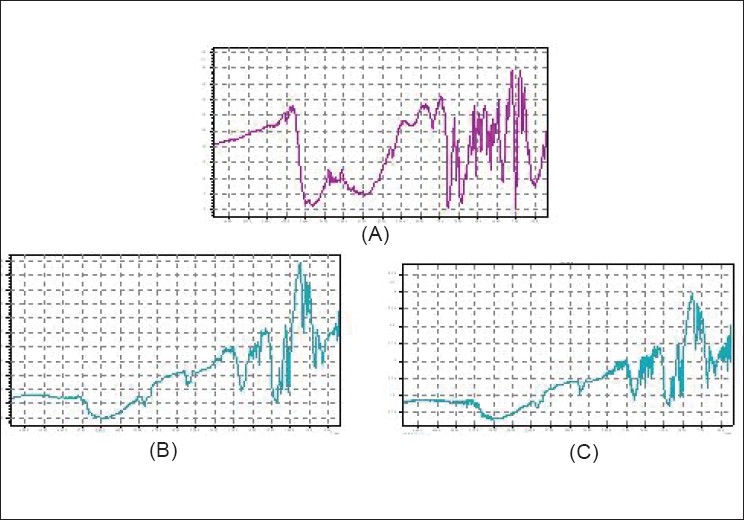
FT-IR spectra of (A) Ondansetron HCl (3,481-3,245.97 cm-1), (B) indion 234 resin (1,674 cm-1), (C) indion-234 resinate

The infrared spectra of drug, indion 234 resin and resinate are depicted in Figure 1. A broad band of bonded -OH of Ondansetron HCl was observed from 3481 to 3245.97 cm^-1^. Indion 234 shows characteristic peaks at 1674 cm^-1^,corresponding to –C=O stretching of aryl acids and due to aromatic C=C stretching. The absence of a peak at 3,481-3,245.97 cm^-1^ in DRC confirms the complexation of the secondary amine group in the drug with resin.

**Figure 2:**
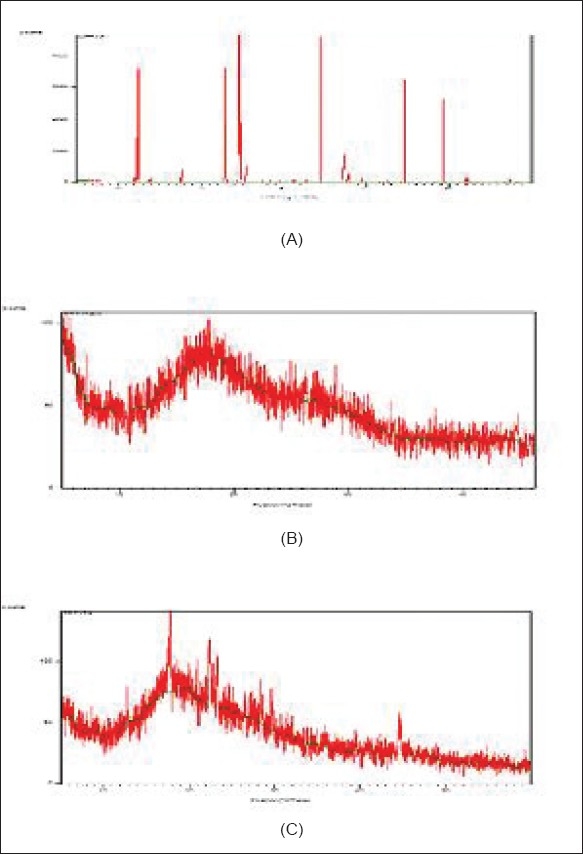
X-ray diffraction pattern of (A) Ondansetron HCl, (B) indion 234 resin, (C) indion-234 resinate

The X-ray diffraction study of drug shows the highly crystalline nature. Resin indion 234 showed an amorphous nature and the resinate showed noncrystalline characteristics. This might be because of entrapment of the drug molecule in the polymer matrix of the resins. From all the evidences, it can be concluded that the drug resinate was a chemical complex [Figure 2]. Studies have shown that the molecules of the entrapped drug change from a crystalline to an amorphous state.[17]

**Figure 3:**
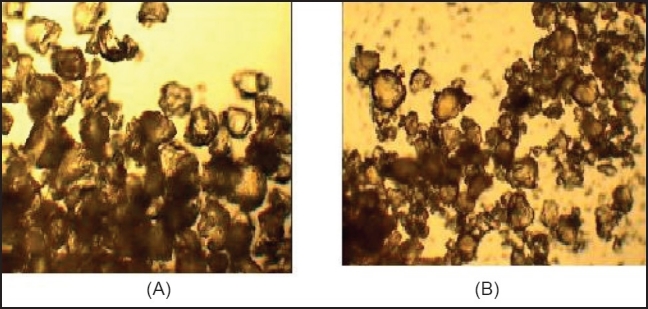
Particle shape of (A) indion 234 resins, (B) indion-234 resinate

The batches of controlled formulations and formulations containing superdisintegrant were designed using higher and lower concentrations of sodium starch glycolate and employing different filler binders and compressed on a tableting machine. For each designed formulation, the blend of drug and excipients was prepared and evaluated for micromeritic properties [Table 3]. The powder was found to be irregular in shape, as shown in Figure 3. Bulk density was found to be between 0.71 and 0.83 g/cm^3^ and the tapped density was found to be between 0.73 and 0.89 g/cm^3^ for all formulations. From density data, % compressibility was calculated and was found to be between 5.55 and 6.66%. Angle of repose was found to be in the range of 30–33. The results showed that the resinates have good flow properties and packing abilities.

**Table 3:** Micromeritic properties of the powder blend*

Formulation	Evaluation parameters						
	Shape	Angle of repose	Bulk density (g/cm^3^)	Tap density (g/cm^3^)	Carr’s index (% CC)	Housner ratio	Flowability
B1	Irregular	32.53 ± 0.38	0.806 ± 0.38	0.862 ± 0.76	6.4 ± 0.52	1.0695	Excellent
B2	Irregular	31.65 ± 0.18	0.7142 ± 0.38	0.7575 ± 0.19	5.716 ± 0.24	1.06	Excellent
B3	Irregular	32.82 ± 0.57	0.8333 ± 0.38	0.8928 ± 0.09	6.6644 ± 0.38	1.0714	Excellent
B4	Irregular	33.00 ± 0.43	0.7552 ± 0.38	0.7812 ± 0.87	5.888 ± 0.35	1.0625	Excellent
B5	Irregular	31.75 ± 0.21	0.7142 ± 0.38	0.7575 ± 0.25	5.7161 ± 0.96	1.060	Excellent
B6	Irregular	30.45 ± 0.87	0.6944 ± 0.38	0.7352 ± 0.31	5.549 ± 0.82	1.058	Excellent
B7	Irregular	32.39 ± 0.64	0.7245 ± 0.38	0.7744 ± 0.54	6.443 ± 0.49	1.0688	Excellent
B8	Irregular	31.72 ± 0.31	0.7178 ± 0.38	0.7674 ± 0.61	6.463 ± 0.73	1.069	Excellent

* Average of three determinations

#### Evaluation of tablets

The prepared batches (B1–B8) of tablets were evaluated for various official and unofficial parameters. Tablets obtained were of uniform weight due to uniform die fill, with acceptable variations as per IP specifications. The hardness, friability and uniformity of content are given in Table 4. Hardness of the tablets for each formulation was between 2.8 and 3.3 kg/cm^2^. Friability below 1.0% was an indication of good mechanical resistance of the tablets. The uniformity of content was found to be 97–102%, which was within the acceptable limits.

**Table 4:** Evaluation of tablets* (batch B1–B8)

Evaluation parameters		Formulations							
		B-1	B-2	B-3	B-4	B-5	B-6	B-7	B-8
Hardness (kg/cm^2^)		2.8±0.6	2.9±0.6	2.9±0.3	3.1±0.6	3.3±0.3	2.8±0.4	2.7±0.8	2.8±0.3
Friability (%w/w)		0.63±0.3	0.75±0.3	0.81±0.8	0.65±0.38	0.67±0.2	0.53±0.3	0.79±0.7	0.44±0.3
Drug content (%w/w)		98.16±0.8	97.05± 0.4	98.37±0.2	99.16±0.9	98.15±0.8	100.16±0.2	102.11±0.3	99.81±0.3
% Weight variation		3.45 ±0.7	4.04 ±0.6	1.43 ±0.4	2.08±1.6	2.78±0.5	2.24 ±0.8	3.87±3.9	3.18±0.6
Water absorption ratio		76.25±0.4	66.36 ±0.7	88.17±0.3	99.36±1.4	113.48±0.5	92.42±0.8	99.14±3.9	106.44±0.
Disintegration time (sec)	*In vitro*	60±1.0	51±0.6	30 ±0.6	28± 0.8	24 ±0.4	35 ±0.6	29±1.2	27± 0.9
	*In vivo*	76±0.4	63±0.4	40±0.2	35±0.6	27±0.6	45±0.8	36±0.5	31±1.1

* Results are the mean of 3 observations ± SD.

Sodium starch glycolate is widely used in oral pharmaceuticals as a disintegrant in tablet formulations. Sodium starch glycolate is the sodium salt of a carboxymethyl ether of starch. The water absorption ratio, *in vivo* and *in vitro* dispersion time are given in Table 4. It was revealed that as the concentration of sodium starch glycolate increases, the water absorption ratio increases and the *in vivo* and *in vitro* dispersion time decreases. But, in the formulations containing starlac as filler and binder, it was observed that sodium starch glycolate does not seem to have much significant effect on the water absorption ratio and dispersion time of the tablets. This may be due to the fact that starlac consists of 15% starch and 85% lactose. Starch has a good disintegrating ability and thus enhances water uptake and dispersion ability. In tablets containing microcrystalline cellulose as filler binder, sodium starch glycolate shows a significant effect on water absorption and dispersion time. This may be attributed to the fact that microcrystalline cellulose is a swellable material and its disintegration characteristics in water have been attributed to the capillary action or swelling.[18]

**Figure 4:**
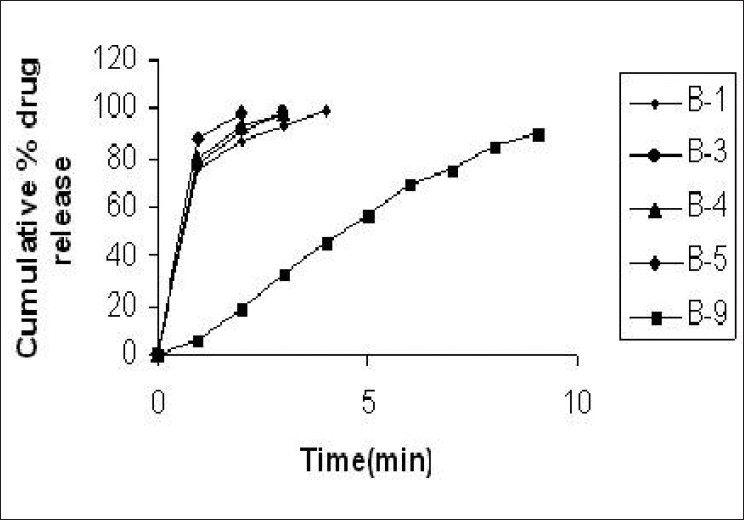
Dissolution profile of control tablet, tablets containing sodium starch glycolate containing starlac as filler binder and the marketed formulation

**Figure 5:**
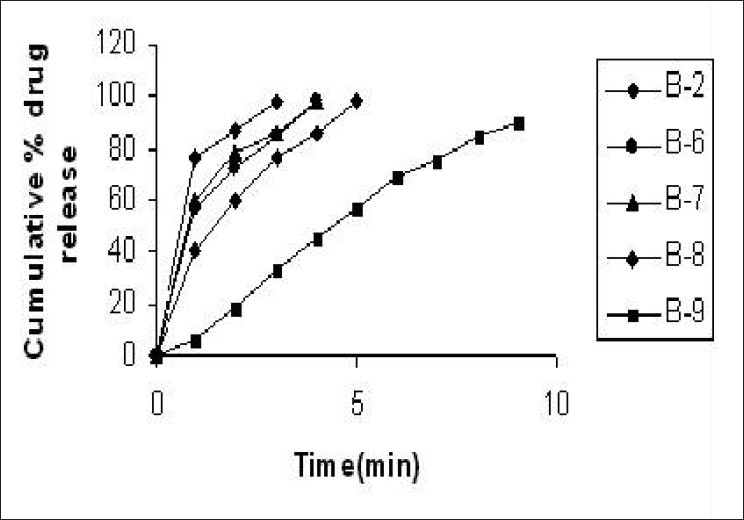
Dissolution profile of control tablet, tablets containing sodium starch glycolate containing MCC as filler binder and the marketed formulation

Dissolution rate was studied for all the tablet formulations and the conventional marketed tablet. For all designed formulations, the tablets show about 98% drug release within 5 min, while the conventional marketed tablet required 9 min for complete release of the drug [Figures 4 and 5]. The formulation B5 containing starlac as filler binder and sodium starch glycolate, showed complete drug release within 2 min [Figure 4] and formulation B8, containing microcrystalline cellulose as filler binder and sodium starch glycolate, showed complete drug release within 3 min [Figure 5]. The drug release from all formulations was too fast as compared to the conventional marketed tablet (B9).

## CONCLUSION

Use of cation exchange resin offers a good method for preparing taste-masked substrates of Ondansetron HCl. Results obtained in this work show that drug–resin complexes effectively masked the bitter taste of Ondansetron HCl. Formulated ODTs showed good release profile, having an additional advantage of complete taste masking. Thus, complexation of Ondansetron HCl with indion 234 increases the acceptability and palatability of the formulated ODTs.
